# Affective super-traits and/or individual patterns: a variable-centered and a person-centered approach of primary emotional aspects of personality

**DOI:** 10.1038/s41598-024-55371-4

**Published:** 2024-02-27

**Authors:** Anita Deak, Orsolya Inhof, Laszlo Nagy, Krisztina Csokasi

**Affiliations:** Faculty of Humanities and Social Sciences, Institute of Psychology, University of Pecs, 6 Ifjusag Street, 7624 Pecs, Hungary

**Keywords:** Affective neuroscience personality scales (ANPS), Latent profile analysis (LPA), Affective typologies, Affective super-traits, Human behaviour, Emotion

## Abstract

Theoretical approaches of personality structure are diverse. We examine the primary emotional aspects of personality as the correspondence of two mainstream constructs: the lexically-based Big Five (BIG5) and the biologically-based Affective Neuroscience Theory (ANT) within two approaches. In the variable-centered approach (VCA), our aim is to identify affective super-traits; while in the person-centered approach (PCA) to uncover latent profile patterns. 240 participants (177 women, 63 men) completed the 112-item affective neuroscience personality scales (ANPS), and the 44-item Big Five Inventory (BFI). We identified four super-traits: Negative emotions (FEAR, SADNESS, Emotional instability), Positive emotions and stimulation (SEEK, Extraversion), Affiliation and social bonds (reversed ANGER, CARE, Agreeableness), Self-regulation (PLAY, Conscientiousness. Based on the VCA, we conclude that the four super-traits represent two main affective tendencies (Positive emotions and approaching, Negative emotions and avoidance), interpersonal (Affiliation) and intrapersonal (Self-regulation) dynamics of personality. As a result of Latent Profile Analysis in the PCA, we explored three latent groups with different patterns of primary emotional traits based on their responsiveness (Highly emotional, Balanced, Low emotional). Our findings provide a holistic approach to emotional aspects of personality, and might have further implications for clinical psychology, neuroscience, and cross-cultural studies on emotions.

## Introduction

In the history of personality psychology diverse theories have competed. They have offered different perspectives focusing on psychodynamic, humanistic, cognitive, or biological aspects^1^. The coexistence of these diverse perspectives enables researchers to see personality in an integrative manner. Undeniably one of the most popular and widely used ways to describe personality differences is the trait approach^2,3^. Conceptual developments such as the trait theory^4^ and statistical methods such as the multiple factor analysis^5^ resulted in some influential personality models postulating that traits can be categorized within a limited number of domains. Besides the currently dominant Five-Factor Model (FFM) (or the Big Five/BIG5/) of Emotional instability, Extraversion, Openness, Agreeableness, and Conscientiousness^6^, several measurement tools have been developed such as the NEO Inventories^7^ and the Big Five Inventory (BFI)^8^. Although, the FFM is well-known and internationally recognized, some critics pointed out its descriptive and phenomenological manner^9^. They argue that the five dimensions merely describe behavior without explaining them and they are not underlying causes of behavior but mere epiphenomena that emerge from evolving patterns of behaviors. Nevertheless, the biological basis of the BIG5 has not been integrated into the original model. Later, evidence from behavior genetics, cross-cultural comparisons and twin studies have made it clear that traits are heritable and grounded solely in biology, but currently little is known about its nature. Researchers claim that trait expression is shaped by the psychological environment, but our understanding is limited to the neurohormonal, or neuroanatomical systems involved.

Although biopsychological-oriented theories (for summary see Supplementary Table 1) identify biological basis of personality, such as the arousability as the function of the ascending reticular activating system (ARAS), neurotransmitter (e.g., dopaminergic and serotonergic) systems, brain structures and networks responsible for motivated behavior (e.g., approach, inhibition), the integrated picture about the biological basis of individual differences in behavior, motivation, emotion, and cognition was provided by a new subfield in neuroscience, namely personality neuroscience^10,11^.

Personality neuroscience is an emerging field that locates the proximal sources of personality in the brain and traces back the brain mechanisms to distal sources in genes-environment interactions. In contrast to the classical personality psychology approach that describes how people differ from each other, personality neuroscience aims to answer not only how, but why they differ from each other. It uses various methods such as neuroimaging (e.g., fMRI, PET), molecular genetics, electrophysiological techniques (e.g., EEG), measurement of electrodermal activity (EDA) or psychopharmacological manipulation to understand the biological sources of personality dimensions. It applies and combines several methods to investigate psychological constructs at both behavioral and neuroscience levels to provide a holistic view^10,11^.

Recent findings in the field of personality neuroscience have identified a stable higher-order factor solution of personality traits^10–13^. Studies of the correlations among the BIG5 traits suggest a two-factor higher-order factor solution, where Emotional instability (reversed), Agreeableness, and Conscientiousness mark the Stability factor, whereas Extraversion and Openness/Intellect mark the Plasticity factor. Plasticity and Stability as higher-order factors (meta-traits) refer to general patterns of behavior, experience, and basic tendencies^14^. Stability appears to represent a general tendency to maintain stability, to regulate or restrain potentially disruptive emotion and behavior, whereas Plasticity appears to represent a general tendency to explore and engage with possibilities and with novelty^11,14^. The main function of Stability is to protect goals, interpretations, and strategies from disruption by impulses; whereas Plasticity promotes exploration to create new goals, interpretations, and strategies^15^. Evidence is accumulating to suggest that Stability is related to serotonin, whereas Plasticity may be related to dopamine^10^.

In the last decade, Panksepp’s Affective Neuroscience Theory (ANT) is in renaissance among biologically based personality models. It has been relevant for personality neuroscience, as well, because it states that the emotional and motivational systems in the brain serve as survival tools^16^. According to the ANT, Panksepp^17^ defines six primary emotional systems (positive: SEEKING, PLAY, CARE; negative: ANGER, SADNESS, FEAR), and highlights them in the context of the FFM^18,19^. This primary-process emotional grounding (e.g., primary emotions or primary emotional traits) influences human personality in a bottom-up manner through the function of subcortical neural networks and contributes to a better understanding of the biological universality of personality and may explain the limited number of factors^20^.

The affective neuroscience personality scales (ANPS)^21^, the measurement tool based on ANT, were developed to serve as a template for emotional personality assessment, also were hypothesized to provide affective underpinnings to the FFM^19^. In Panksepp’ view, affective neuroscience suggests an enhanced neuroscientifically premised personality research model. It provides a putative physiological basis for the FFM traits, helps the FFM escape circular reasoning by suggesting brain mechanisms that can be manipulated to influence the expression of personality. Moreover, it contributes to advance personality theory from trait and situationist approaches to interactionist and ontogenetic personality models. Based on the gap derived from the poorly discovered biological basis of FFM, and the strong biological and evolutionary background of the ANT, in this paper we suggest that the integration of a biologically based system (ANT) and a descriptive behavioral trait-like system (FFM) can contribute to a deeper understanding of personality structure.

Former studies have found evidence on the emotional aspect of some of BIG5 factors. Based on both self-report measures^22^ and brain imaging studies^23^, for example, Emotional instability has been related to negative emotional experiences, while Extraversion has been more linked to positive emotions. However, to our knowledge, our study is the first to investigate the associations in a complex and systematic way.

The correlations between the BIG5 and the ANPS domains show similar patterns across samples and measurements^24^. A recent meta-analysis^18^ has shown that high SEEKING relates to high Openness to Experience, high PLAY to high Extraversion, high CARE/low ANGER to high Agreeableness and high FEAR/SADNESS/ANGER to high Emotional instability. Conscientiousness seems to be less directly related with the subcortical primary emotions and likely is the most cognitive/cortical personality construct out of the BIG5.

### Two approaches of personality: a variable-centered versus a person-centered approach

Many researchers follow the variable-centered approach that assumes homogeneity in a population. According to this approach average scores are calculated, and associations are examined between different dimensions and constructs (e.g., high Emotional instability is associated with higher risk of personality disorders; low Agreeableness is a risk factor for substance abuse), furthermore covariations are identified. For example, the FFM provides a structure in which most personality traits can be classified^9^. This structure arises because traits covary. People who are sociable and assertive tend also to be cheerful and energetic; they have high scores on the Extraversion factor, which is said to be defined by sociability, assertiveness, cheerfulness, and energy. However, people who are sociable and assertive may or may not be intellectually curious and imaginative. These latter traits belong to the separate Openness factor.

In contrast, the person-centered approach is less common in personality research^25^. It has its roots in personality typologies, as it identifies qualitatively different sub-groups (i.e., profiles or types) within the population. One advantage of the person-centered approach is that the typologies (i.e., sub-groups) are empirically derived, and the existence of the sub-population is statistically tested. For example, typologies could be created by classifying people obtaining the upper 20% scores on the variables of interest in one profile, those obtaining the lower 20% scores in a second profile, and those obtaining intermediate scores in a third profile. However, profiles can change if the cut-off scores are modified (e.g., from 20 to 15%). Thus, models are necessary to be tested to verify if one or more sub-groups assumptions fits the best to the data.

Previously, in the field of emotional reactivity and emotional regulation, different typologies appeared^26–28^. For example, individuals were classified by how they react to emotional cues, especially negative, unpleasant ones. Some people are very sensitive and alert, moreover they continuously monitor the environment (e.g., sensitization/monitoring/vigilance), others seem to have higher perceptual threshold in responding to negative stimuli, and they tend to avoid (or keep a distance from) stressful stimuli or situations (e.g., repression/blunting/avoidance). Consequently, the person-centered approach enables scientists to create different profiles to describe the different sub-populations.

The majority of recent ANPS studies follow the variable-centered approach^25^ that (1) assumes homogeneity in a population by describing the average of ANPS dimensions; (2) focuses on the associations between one (or more) ANPS dimension(s) and one (or more) other construct(s) (e.g., SEEKING and depression)^29^; and (3) explores the role of one (or more) ANPS dimension(s) using regressions or Principal Component Analysis^30^. Orri et al.’s study^25^ was the first to use a person-centered approach with ANPS assuming heterogeneity in the population based on Panksepp’s biologically based six primary emotional traits. The exploration of typologies defined by a person-centered approach can contribute to considering personality as a dynamic system of evolutionary adaptive emotional traits. Three profiles were characterized among men and women, respectively, conducting Latent Profile Analysis (LPA) in a cross-sectional and a longitudinal study involving two samples (French college students from Paris area in the cross-sectional study, and young Canadian parents in the longitudinal study). In the Canadian sample the first profile was characterized by low levels of negative emotions and average levels of positive emotions (Low negative emotions profile), a second profile characterized by average levels of emotionality (Balanced profile), and a third profile characterized by high levels of both positive and negative emotionality (High emotional profile). In the French sample, the same three profile was found among men. However, only the Low negative profile and the High emotional profile was identified for women. The second profile was characterized by an imbalance of low positive emotions and medium level of negative emotions. Although the typologies were similar between men and women, the mean scores differed for both genders (e.g., women scored higher on CARE, FEAR and SADNESS). These results were in line with previous studies using a variable-centered approach that found significant gender effect on the ANPS dimensions^31^. Therefore, the classification conducted in the person-centered approach (using LPA) did not change the personality differences of men and women that have been found consistent with previous literature. The discrepancy between the second profile among French and Canadian women was explained by socio-demographic factors such as older age and parental family status for Canadian women. In addition, the ANPS profiles showed stability for both genders over four years. In conclusion, Orri et al.’s study was the first to identify latent affective personality profiles in a neuro-evolutionary theoretical framework measured by the ANPS.

## Aims, research questions and hypotheses

As personality theories should not be limited to a particular domain of information-processing, but must consider individual differences in affect, behavior, and cognition, as well as how these different domains are integrated and interact^32^, we adapt the integrative and multiple-perspective framework of personality neuroscience^13^, an emerging and promising subfield in neuroscience. The goal of this study is twofold: 1. In a variable-centered approach, the ANT would not only contribute to understand the biological basis of the BIG5 factors, but we investigate them in an evolutionary framework. Thus, we examine the relationship between the five domains of the descriptive FFM and the six, biologically based emotional traits of the ANT (Research aim 1; RA1) to confirm the correlations between BIG5 and ANPS in a Hungarian sample. Furthermore, in a variable-centered approach and in line with recent findings of personality neuroscience about two meta-traits^14,15^, we aim to identify meta-traits (or super-traits) that consist of the six primary emotional traits, and the five main personality domains to explore a higher-order structure behind the affective aspects of personality traits (RA2); 2. in a person-centered approach our goal is to explore individual differences and to identify the fewest number of homogenous subgroups of individuals (latent classes or latent profiles) within the healthy young adult sample (RA3).

Three groups of research questions (RQ) are as follows: RQ1: What pattern of PETs can be associated with the BIG5 domains? What are the significant primary emotional traits that predict one or more BIG5 domains? RQ2: How many affective super-traits can be identified if the BIG5 domains and the six PETs are included in one integrative model? Do these affective super-traits correspond to previous findings of a higher-order factor solution? RQ3: How many latent groups can be explored based on the different patterns of the six PETs? How do these latent sub-groups differ in the BIG5 domains?

### Variable-centered predictions (H1)

Based on the correlations between ANPS scales and the BIG5 facets in former ANPS-validation studies^20,33,34^, we hypothesize that PETs in ANPS are associated with BIG5 personality domains in a Hungarian sample, namely (H1a) high SEEKING relates to high Openness; (H1b) high PLAY relates to high Extraversion; (H1c) high CARE and low ANGER relate to high Agreeableness; (H1d) high FEAR, SADNESS and ANGER relate to high Emotional instability.

### Affective super-traits (H2)

According to DeYoung’s concept of meta-traits referring to basic psychological functions of personality^14^, we expect to identify two super-traits. We hypothesize that (H2a) the one super-trait consists of negative PETs (FEAR, ANGER, SADNESS), and Emotional instability; and (H2b) the other super-trait contains positive primary emotional traits (SEEKING, PLAY, CARE), Extraversion and Openness.

### Person-centered predictions (H3)

Based on previous findings among French and Canadian samples^25^ and theoretical assumptions about typologies of emotional reactivity and information processing, we hypothesize the existence of three profiles among men and women, respectively. We expect three qualitatively different profiles as follows (H3): high positive emotional traits with low negative emotional traits (Profile 1), a balanced profile where the positive and negative scores are similar (Profile 2) and a highly emotional profile with high scores on both positive and negative ANPS scales (Profile 3).

## Methods

### Participants

240 participants (177 women, 63 men) filled in the electronic version of the questionnaire battery. The mean age was 27.84 years (SD = 12; min.: 18, max.: 75). Regarding the sociodemographic background, they have spent 15 years in education on average; most of them are still university students; have been living in urban conditions (e.g., in a town or a city), and evaluated their financial situation satisfying or good. This study was approved by the United Ethical Review Committee for Research in Psychology (EPKEB; reference number 2017/90). All procedures were in accordance with the ethical standards in the 1964 Declaration of Helsinki. All participants signed informed consent after reading a statement of the study’s purpose, procedure, and confidentiality.

### Measures

#### Affective neuroscience personality scales (ANPS)

Participants filled in the Hungarian version^35^ of the 112-item ANPS 2.4^21^. It has three positive emotional scales (PLAY, SEEK, CARE), three negative emotional scales (SADNESS, ANGER, FEAR) and Spirituality. Responses are collected on a four-point Likert scale (1 = strongly disagree; 4 = strongly agree). The Cronbach alfa values were acceptable or relatively high in the current sample (PLAY: 0.84; SEEK: 0.77; CARE: 0.73; SADNESS: 0.77; ANGER: 0.85; FEAR: 0.90). As in this study we investigate the role of primary emotional traits, we do not report Spirituality scores, but only the six emotional scales.

#### Big Five inventory (BFI)

We used the Hungarian version of the 44-item BFI^8^ to measure the five domains of personality: Openness to Experience, Conscientiousness, Extraversion, Agreeableness, and Emotional instability. Participants indicate their responses on a five-point Likert scale (1 = strongly disagree; 5 = strongly agree). The reliability values are good for the current sample (Cronbach alfa values: 0.73–0.89).

### Procedure

We used the Gorilla Experiment Builder (www.gorilla.sc)^36^ to collect questionnaire data electronically. It took 25–30 min to complete the questionnaire battery. First, we provided a detailed description of the study, then participants gave informed consent. Questionnaires appeared after the acceptance of the consent form and the demographic data sheet (respondent’s gender, age, place of residence, highest level of education, years spent in education, occupation, hobbies, mother’s highest level of education and subjective evaluation of financial situation).

### Statistical analysis

We used Jamovi statistical software (Version 2.2) for data analysis^37^. In the variable-centered approach (RQ1, H1), firstly, descriptive, and correlational analyses were used, and series of multiple regression analyses were conducted to identify whether the scores on the six emotional scales of ANPS are related to the BIG5 personality dimensions, respectively.

Secondly (RQ2, H2), we used principal axis factor analysis with varimax rotation to identify affective super-traits based on eleven variables (i.e., six ANPS scales and five BFI dimensions). We tested the classical two-factor model for positive and negative emotionality, and an alternative model.

In the person-centered approach, thirdly (RQ3, H3), we conduct latent profile analysis (LPA) to test whether LPA models with 2 to 5 latent classes are fitted the best for men and women, respectively. LPA is a specific case of a finite mixture model^38^ to investigate population heterogeneity by sorting cases into homogeneous latent subgroups of respondents (latent classes) that are more similar to each other than other subgroups. It allows researchers to investigate individual differences that explain variability among people, that is whether the observed pattern of responses is best explained by a few set dimensions or domains that distinguish ‘types’ of responders. We decided to explore ANPS profiles in men and women separately because significant gender differences in the ANPS dimensions have been regularly found in the literature. The best profile model is selected using fit indices, however, theoretical grounding and/or previous research must be considered when deciding about the number of models and interpreting the results to avoid misinterpretation of the empirical results. We assessed the best-fit indices including (1) Akaike Information Criterion (AIC); (2) the Bayesian Information Criterion (BIC); (3) the sample size-adjusted BIC (SABIC); (4) the entropy that indicates the accuracy which models classify participants into the most likely class; (5) the bootstrap likelihood ratio test (BLRT). A smaller value of AIC, BIC, and SABIC, a low and significant p-value of BLRT as well as, a higher value of entropy (range 0–1) indicated a better-fitting model (For more information on the fit indices see the Supplementary material from Miettunen et al.^38^). A series of ANOVA was conducted with Bonferroni post-hoc correction to compare the means of the five BFI dimensions among the three profile groups defined by the LPA for women and men, respectively.

## Results

### Variable-centered approach: relationships between ANPS and BFI (H1)

A series of multiple regression analyses was conducted to investigate the pattern of PETs in association with the BIG5 personality dimensions, respectively. The means, standard deviations, and correlations can be found in Table 1.

**Table 1:** Descriptive statistics and correlations between questionnaire variables.

	SEEK	FEAR	CARE	ANGER	PLAY	SADNESS	Mean (SD)
Extraversion	0.43***	− 0.39***	0.25***	− 0.09	0.42***	− 0.15*	27.73 (6.63)
Agreeableness	0.27***	− 0.14*	0.51***	− 0.53***	0.26***	0.06	32.00 (5.88)
Conscientiousness	0.30***	− 0.33***	0.21**	− 0.27***	− 0.08	− 0.24***	32.67 (7.30)
Emotional instability	− 0.14*	0.76***	− 0.07	0.55***	− 0.22***	0.54***	23.81 (6.86)
Openness	0.44***	− 0.09	0.23***	− 0.07	0.22***	− 0.02	37.77 (7.13)
Mean (SD)	44.26 (6.15)	39.03 (9.08)	43.58 (6.38)	35.41 (8.04)	42.21 (7.43)	39.02 (6.89)	

*p < 0.05, **p < 0.01, ***p < 0.001.

When all six PETs were included in a regression model, they were significant predictors of the BIG5 personality dimensions, respectively. Different combinations of PETs contributed the different personality dimensions (Table 2).

**Table 2:** Regression results using the six ANPS scales and the BIG5 dimensions, respectively Primary.

Predictor	Unstandardized b	SE	Standardized β	t	p
Openness					
SEEK	0.46	0.08	0.40	6.08	< 0.001
FEAR	− 0.05	0.07	− 0.06	− 0.72	0.47
CARE	0.08	0.08	0.08	1.06	0.29
ANGER	0.03	0.06	0.03	0.52	0.60
PLAY	0.05	0.06	0.06	0.86	0.39
SADNESS	− 0.05	0.09	− 0.05	− 0.56	0.57
Conscientiousness					
SEEK	0.38	0.07	0.32	5.23	< 0.001
FEAR	− 0.23	0.06	− 0.28	− 3.60	< 0.001
CARE	0.27	0.08	0.24	3.61	< 0.001
ANGER	− 0.07	0.06	− 0.08	− 1.32	0.19
PLAY	− 0.34	0.06	− 0.35	− 5.73	< 0.001
SADNESS	− 0.16	0.08	− 0.15	− 1.99	0.05
Extraversion					
SEEK	0.35	0.06	0.33	5.77	< 0.001
FEAR	− 0.33	0.05	− 0.45	− 6.12	< 0.001
CARE	0.11	0.06	0.10	1.69	0.09
ANGER	0.14	0.05	0.16	2.86	0.01
PLAY	0.18	0.05	0.21	3.62	< 0.001
SADNESS	0.05	0.07	0.06	0.78	0.44
Agreeableness					
SEEK	< 0.01	0.05	< 0.01	0.05	0.96
FEAR	− 0.03	0.04	− 0.05	− 0.70	0.48
CARE	0.46	0.05	0.49	9.05	< 0.001
ANGER	− 0.30	0.04	− 0.41	− 8.04	< 0.001
PLAY	0.03	0.04	0.03	0.64	0.52
SADNESS	0.05	0.05	0.06	0.94	0.35
Emotional instability					
SEEK	− 0.10	0.05	− 0.09	− 2.08	0.04
FEAR	0.43	0.04	0.57	10.24	< 0.001
CARE	− 0.05	0.05	− 0.05	− 1.08	0.28
ANGER	0.23	0.04	0.27	6.26	< 0.001
PLAY	< 0.01	0.04	< 0.01	0.06	0.96
SADNESS	0.12	0.05	0.12	2.26	0.02

High FEAR, ANGER and SADNESS with low SEEK significantly predicted Emotional instability, F(6,233) = 75.27, p < 0.001. The adjusted R^2^ value was 0.651 indicating that 65% of the variance for Emotional instability can be explained by primary emotions.

Regarding Agreeableness, high CARE and low ANGER were significant predictors, F(6,233) = 44.82, p < 0.001, and the model explained 52% of the variance (adjusted R^2^: 0.524).

SEEK, FEAR, ANGER and PLAY significantly predicted Extraversion, F(6,233) = 26.82, p < 0.001. The adjusted R^2^ value was 0.393. This indicates that 39% of the variance in Extraversion is explained by the model built up from the primary emotions. That is, low FEAR, high SEEK, ANGER and PLAY predicts Extraversion.

As predictors of Conscientiousness, SEEK, FEAR, CARE, PLAY and SADNESS played a significant role, F(6,233) = 18.45, p < 0.001. High scores on SEEK and CARE, as well as low scores on FEAR, ANGER, PLAY and SADNESS explain 31% of the variance.

Regarding Openness, the model including six primary emotions was significant, F(6,233) = 10.28, p < 0.001 (adjusted R^2^: 0.189). However, only high SEEK has a significant predicting role in the model.

### Affective super-traits: a higher-order factor structure (H2)

We conducted principal axis factor analysis with varimax rotation to assess the underlying structure for personality based on eleven variables (i.e., the six emotional scales of ANPS and the BIG5 dimensions). We tested both the two-factor model^14^ and an alternative model.

After rotation, the two factors explained 53% of the variance. The first factor of negative affects accounted for 27%, and the second factor of positive affects accounted for 26%. We tested an alternative model where the number of factors were not limited. Four factors were identified (Eigenvalue > 1) and explained 75% of the total variance. After rotation, the first component consisted of five variables, such as, FEAR, SADNESS, and Emotional instability. They explained 25% of the variance. The second component consisted of SEEK, Extraversion and Openness. They accounted for 20% of the variance. In the third component, we found Agreeableness, CARE and ANGER with negative loading. They accounted for 20% of the variance. The fourth factor consisted of two variables: PLAY and Conscientiousness with negative loading. They accounted for 10% of the variance. Table 3 displays the variables and factor loadings after rotation for the two-factor model and for the alternative model of four factors.

**Table 3:** Results from factor analyses including eleven variables to test the two-factor model and an alternative model with four factors for super-traits.

	Factor loading for the two-factor model		Factor loading for the alternative model with four factors			
	1	2	1	2	3	4
FEAR	0.896		0.904			
Emotional instability	0.878		0.819			
SADNESS	0.798		0.846			
ANGER	0.548				− 0.709	
Conscientiousness	− 0.499					− 0.717
Extraversion		0.563		0.737		
CARE		0.800			0.724	
SEEK		0.733		0.788		
Agreeableness		0.694			0.883	
PLAY		0.534		0.448		0.737
Openness		0.515				

### Person-centered approach: exploration of latent profiles (H3)

We tested four models with two to five groups for men and women, respectively. Fit indices are shown in Table 4, with the number of participants in each profile. For men, the AIC and BIC values are the lowest in the three-profile model. Although the SABIC value decreased and the entropy value increased from the two-profile to the five-profile models, the p value of BLRT is not significant in the four-profile model, thus the five-profile model has no additional contribution. The entropy value for the three-profile model is 0.76 which is acceptable. For women, the AIC and SABIC values decreased, the entropy values increased from the two-profile to the five-profile models. Although the five-profile model had the best fit indices, the number of participants in each subgroup is unequal, and extremely low in some cases (n = 6), thus comparison of subgroups’ scale means might not be valid. The fit indices for both the four-profile and the three-profile models are similar and acceptable, and theoretically meaningful. Due to conceptual considerations, we selected three profiles both for men and women.

**Table 4:** Fit indices for the latent profile analysis of six ANPS scales for men and women, respectively.

	Model	LL	AIC	BIC	SABIC	Entropy	BLRT (p)	Class size N (%)				
								P1	P2	P3	P4	P5
Men	Class 2	− 1267.50	2572.99	2613.71	2553.92	0.64	21.54***	21 (33)	42 (66)			
	Class 3	− 1244.14	2540.28	2596.00	2514.18	0.76	46.72***	26 (41)	15 (25)	22 (54)		
	Class 4	− 1238.87	2543.74	2614.47	2510.62	0.78	10.53	16 (25)	15 (24)	8 (13)	24 (38)	
	Class 5	− 1226.37	2532.74	2618.46	2492.59	0.83	25.00*	29 (46)	10 (16)	5 (8)	9 (14)	10 (16)
Women	Class 2	− 3512.81	7063.62	7123.96	7063.79	0.73	124.32**	96 (54)	81 (46)			
	Class 3	− 3472.92	6997.83	7080.41	6998.08	0.77	79.78***	77 (43)	72 (41)	28 (16)		
	Class 4	− 3453.99	6973.99	7078.80	6974.30	0.80	37.85***	68 (38)	67 (37)	17 (10)	25 (14)	
	Class 5	− 3444.81	6969.61	7096.66	6969.99	0.81	18.37*	63 (36)	66 (37)	17 (10)	25 (14)	6 (3)

*LL* loglikelihood, *AIC* Akaike information criterion, *BIC* Bayesian information criterion, *SABIC* sample size-adjusted BIC, *BLRT* bootstrap likelihood ratio test, *N* number of participants in each subgroup.

*p < 0.05; **p < 0.01; ***p < 0.001.

**Figure 1:**
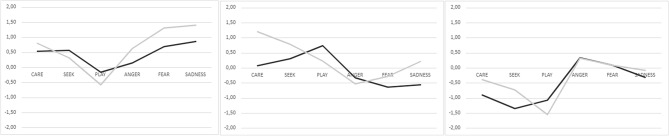
Three profiles explored by latent profile analysis (LPA). Profile 1: High emotional; Profile 2: Balanced profile; Profile 3: Low emotional. The figure shows the profile plots created from Z-scores for men (black lines) and women (gray lines).

Men’s and women’s profiles are presented in Fig. 1. Descriptive statistics, gender differences and pair-wise comparisons are reported in Supplementary Tables 2–7.

Both among men (35%, n = 22) and women (43%, n = 77), Profile 1 (High emotional with hyperactivation strategy) had the highest scores on the negative scales compared to the other two profiles. Profile 2 (Balanced) had similar scores both on the positive and the negative scales (men: 41%, n = 26; women: 41% n = 72). Profile 3 (Low emotional with deactivation strategy) had the lowest values for the positive scales and relatively low on the negative scales (men: 24%, n = 15; women: 16%, n = 28).

Statistically significant difference was found in the five BFI facets among the three groups defined by the three profiles for men and women, respectively. Supplementary Tables 2–7 show the statistical values, means, standard deviations and effect sizes. In both men and women, individuals in the balanced profile (P2) reported higher scores on Extraversion, Agreeableness, Conscientiousness, Openness, and lower scores on Emotional instability compared to the other two profiles. The mean differences between high emotional (P1) and low emotional (P3) groups’ mean scores, however, were smaller among men, and greater among women.

## Discussion

The multiple and integrative perspective of personality neuroscience, an emerging and promising field, focuses on explanation rather than description of personality. The FFM is a widely accepted, internationally recognized theory, but as a descriptive model of personality it shows how individuals differ from each other, and its explanatory power is weak on why individuals differ from each other. Alternatively, biopsychological theories, for example Panksepp’s ANT^17^, emphasizes the neural background and the evolutionary history of human emotional functioning with the existence of six primary emotional traits. In this study, we suggest that the ANT is suitable to explore the affective aspects of personality traits in an evolutionary framework. Previous studies have shown some affective aspects of FFM, especially the relationships between Emotional instability and negative emotions, Extraversion and positive emotions, however, to our knowledge, our study is the first that takes the personality neuroscience perspective and combines the ANT as an influential biopsychological personality theory with the descriptive FFM to respond three research questions to explore the affective aspects of personality both in a variable-centered and in a person-centered approach. Our aim was (1) to uncover the different patterns of primary emotions that contribute to the main five personality dimensions, (2) to test the classical two-factor model of affects and to identify the higher-order structure of personality, that is affective super-traits, and (3) to investigate individual differences through the exploration of latent subgroups within a population.

### Affective aspects of personality in a variable-centered approach (H1)

The associations between the ANPS scales and the BIG5 dimensions were congruent with the findings of a recent meta-analysis^18^ and different adaptation studies of ANPS^20,33,34,39,40^. The methodological importance of this convergence is that this result supports the validity of the Hungarian version of ANPS^35^. Furthermore, the theoretical importance is that we have contributed to the better understanding of cultural similarities and differences regarding PETs.

RQ1 referred to the associations between primary emotions and personality. Our results have confirmed that primary emotions serve as predictors of the five main personality domains. In line with a recent meta-analysis^18^, we found the most robust results in the case of Emotional instability. We hypothesized that high FEAR, SADNESS and ANGER relate to high Emotional instability (H1d). Our findings point out that the intense presence of negative emotions (FEAR, SADNESS, ANGER) together with a low level of interest and exploration (SEEK) are in association with Emotional instability. In the original publication of ANPS^21^, in the Turkish^40^, Spanish^33^, and Serbian validation study^39^ a moderately to strong association of Emotional instability with SADNESS and FEAR was found, and somewhat weaker with ANGER (except for the Turkish sample where ANGER had the strongest correlation with Emotional instability). According to our results, FEAR and ANGER play the major role in predicting Emotional instability (positively), while SADNESS and SEEK have a minor role; the former increases Emotional instability, the latter decreases it. This inverse association of positive affects with Emotional instability was found also in the French^34^, Turkish^40^, German, and Chinese samples^20^. Besides the strong association between negative affects and Emotional instability in the last two samples, a weak negative correlation was found between SEEK, PLAY and Emotional instability, but the CARE-Emotional instability relationship was positive and significant only in the German and French samples.

We hypothesized that high CARE and low ANGER relate to high Agreeableness (H1c). Former results from the French^34^, Spanish^33^, Turkish^40^, German, and Chinese samples^20^ indicate that Agreeableness is associated positively with the three positive ANPS scales and negatively with ANGER. In the Serbian sample^39^, the correlation was significant only with CARE (positively) and ANGER (negatively). In line with a recent meta-analysis^18^, we found that CARE positively and ANGER negatively play a crucial role as predictors of Agreeableness. Thus, we support the proposal that the dimension of Agreeableness may integrate CARE and ANGER^21^, where feelings related to CARE would be situated at the positive pole and feelings related to ANGER at the negative pole.

Regarding Extraversion, we hypothesized that high PLAY relates to high Extraversion (H1b). We found that high levels of positive emotions such as SEEK and PLAY, a high level of ANGER and a low level of FEAR contribute to predicting Extraversion. Unlike the meta-analysis^18^ and the US^21^, French^34^, Spanish^33^, Serbian^39^, German, and Chinese samples^40^ where Extraversion correlated with PLAY, we suggest a more sophisticated picture of the role of additional emotional traits (i.e., high ANGER and low FEAR). Weak or moderately strong negative correlations with one or more ANPS negative scales were found also in the French^34^, Turkish^40^, Serbian^39^, German, and Chinese samples^20^. Our results are very similar to the Serbian results^39^, namely Extraversion is associated with high SEEK, high PLAY and low FEAR, but without low SADNESS, and the lack of correlation with ANGER (in the Serbian sample).

Based on the meta-analysis, Conscientiousness has been found less directly related with primary emotions but might go along with a top-down control of them. It showed a weak positive correlation with SEEK and CARE in the Spanish^33^, Turkish^40^, Chinese, and German samples^20^ weak negative correlations with FEAR, ANGER and SADNESS in the original US study^21^, and with FEAR and SADNESS in the Serbian^39^ and Chinese samples^20^. No correlation was found with negative emotional traits in the Turkish^40^ and in the German samples^20^. In the French sample^34^, however, FEAR and CARE showed a weak positive correlation with Conscientiousness, while PLAY correlated negatively with it. Although our study may not provide an integrative solution of these previous contradictory findings, we claim that beside the role of SEEK and CARE as positive predictors, there is an inverse contribution of PLAY and the three negative emotions.

We hypothesized that high SEEKING relates to high Openness (H1a). In line with the findings of the meta-analysis^18^, our results support the evidence that SEEK relates to Openness. Unlike the Spanish study^33^, where all ANPS scales (except for ANGER) correlated positively with Openness, we found SEEK to be the only significant predictor. However, this finding is in line with the original US study^21^, where SEEK was the only scale that correlated with Openness. Besides with SEEK, a weak positive correlation was found with CARE and SADNESS in the German sample, with CARE and PLAY in the Chinese sample^20^, and a weak negative with FEAR and a weak positive with PLAY in the French sample^34^. Openness correlated positively with all three positive scales in the Serbian^39^ and Turkish samples^40^, moreover negatively with ANGER also in the Turkish study, but not in the Serbian.

Besides the main facets of personality, we successfully tested the validity of ANPS scales with positive and negative affects. The Spanish validation study^33^ is the only in which the two scales of the PANAS questionnaire were added to an exploratory factor analysis together with the six ANPS scales to test the bifactorial structure of affects. Unlike their results, namely the PANAS Negative Affect scale, FEAR, ANGER and SADNESS were identified as the negative affect factor; PANAS Positive Affect, PLAY, CARE and SEEKING formed the positive affect factor, we suggest that specific patters of primary emotional traits constitute the positive and negative affects, respectively. Positive affects are associated with more interest and explorative behavior (SEEK), social bonds with others and caring for others (CARE) and a low level of anxiety (FEAR). Negative affects, on the other hand, are connected to frustration (ANGER), isolation (SADNESS), anxiety (FEAR) and a weak motivation to find alternative solutions or new situations (SEEK).

### Affective super-traits (H2)

We expected the existence of two super-traits (RQ2); however, we also tested an alternative solution. We hypothesized that (H2a) the first super-trait consists of negative PETs (FEAR, ANGER, SADNESS), and Emotional instability; and (H2b) the second super-trait contains positive primary emotional traits (SEEKING, PLAY, CARE), Extraversion and Openness.

Our two-factor model confirmed former findings^33,34^ that Emotional instability, FEAR, ANGER and SADNESS represent the factor of negative emotions, whereas the other factor consisted of Extraversion, Agreeableness, Openness, SEEK, CARE and PLAY. Although our bifactorial structure solution strongly matches the results of the Spanish^33^ and the French^34^ adaptation study, there are some minor differences. Unlike Abella et al.’s work^33^, where Conscientiousness did not belong to any of the factors, in our study it was represented negatively in the factor of negative emotions.

In a Turkish sample, however, four factors have been identified after the exclusion of Consciousness^40^. The factor for negative emotions in our study is fully matches with Factor 1 (Emotional instability) in the Turkish study, and partly matches with Factor 2 (Agreeableness). Different factor-structure appeared regarding the other two factors. Instead of Extraversion (Extraversion and PLAY in Factor 3) and Openness to experiences (Openness and SEEK in Factor 4), in the Hungarian PLAY positively and Conscientiousness loaded the same factor, whereas Extraversion loaded with SEEK. We conclude that there are cultural similarities in the first two factors and a stronger impact of culture is presented in the last two factors. However, it might be the case that the slightly different questionnaire translations (Turkish and Hungarian) caused the differences.

The cross-cultural interpretation of the higher-order factor structure of the ANPS is in line with recent research trends^41^. Cross-cultural affective neuroscience as a new research field aims to investigate the cultural influence on basic affective systems, that is the similarities and differences how social-cultural factors like mothering style, family models, social interactions regulate the subcortical affective systems. This influence can be investigated by observing the cultural variations either at the level of lower-order factor structure (i.e., the six emotional scales of ANPS) or at a higher-order factor structure (e.g., meta-traits). Although the ANPS has been translated into several languages and it has the potential to assess both the universal genotypes and the culturally specific phenotypes, the universal findings were more emphasized. Future research directions are expected to focus more on the differences.

The two meta-traits of positive emotions and negative emotions computed from the ANPS show only the valence of the subjective affective experience (e.g., positive–negative, pleasant-unpleasant). The two meta-traits of Plasticity and Stability computed from the BIG5 show a broader picture of the main functions of personality to adapt to the constantly changing environmental conditions. As one of our aims was to explore a higher-order factor structure behind the affective aspects of personality traits, we tested an alternative model. Based on the results of an exploratory factor analysis, we suggest an alternative four-factor solution that has stronger explanatory power compared to the two-factor model. In the four-factor model, the first factor represented negative emotions and avoidance. It consisted of FEAR, SADNESS, and Emotional instability. This result corresponds with former findings^42^ that Emotional instability and BIS predicted increased fear and sadness reactivity. The second factor represented positive emotions, exploration and approach including SEEK, Openness, and Extraversion. In line with our results, previous correlational analyses^43^ showed that extraversion was positively related to the frequency, intensity, and duration of positive emotions whereas emotional instability was positively related to the frequency and duration of negative emotions. The duration of positive emotions proved to be the strongest predictor of extraversion whereas the frequency of negative emotions is the strongest predictor of emotional instability. The third factor is an interpersonal construct (Affiliation) where CARE indicates positive attitude, prosocial behavior, and interest towards others, while ANGER might regulate social distance from peers through negative emotion. Moreover, we suggest that low ANGER (besides high CARE) indicates that the lack of impulsivity contributes to positive social interactions. The fourth factor is interpreted as an intrapersonal construct. As high PLAY and low Conscientiousness belonged to this factor, we suggest that it might be responsible for the flexibility structure and the resilient function of personality through self-regulation and self-directedness. We agree that Conscientiousness may be culturally modulated^40^ as it refers to the way how social rules and norms reinforce individual’s behavior either in an individualistic or a collectivistic context.

In sum, the two-factor solution of affective super-traits were similar to former empirical findings from other cultures; however, the four-factor solution including two addition factors (intra-and interpersonal factors) proved to match better to current research trends in cross-cultural affective neuroscience about the socio-cultural influence of subcortical emotional functions. Further studies should be conducted to clarify this effect.

### Latent profiles in a person-centered approach (H3)

In contrast to the variable-centered approach, the person-centered approach assumes heterogeneity in the population and uncovers latent sub-groups in which individuals present similar affective patterns. LPA is suitable to select from profile models with different number of latent sub-groups. Selection of the best profile model based exclusively on the fit indices without theoretical grounding or previous empirical findings may lead to misinterpretation of the results. Thus, our prediction in the person-centered approach was based on previous findings of Orri et al.^25^ (RQ3). We expected three qualitatively different profiles (H3) in both genders separately because significant gender differences in the ANPS dimensions have been regularly found in the literature, as well as in the current sample. Both among men and women, we identified the three classes as follows: High emotional subgroup (Profile 1), Balanced subgroup (Profile 2) and Low emotional subgroup (Profile 3).

Profile patterns showed similarities with previous findings^25^ indicating that affective typologies might differentiate among individuals based on their relative balance of positive and negative emotional experiences. It is in line with psychopathological implications published in the early version of ANT^44^, that the unbalance of primary affective systems (either underactivation or overactivation) might be risk factors for psychopathological disorders. The results of our study are not enough to draw conclusions like this, we only claim that affective typologies upon the balance or unbalance of negative and positive emotions can open new directions for further research using the ANPS.

Additional findings between the ANPS profiles and emotion regulation strategies^25^ indicated that individuals with low negative emotions reported themselves to be better able to understand and regulate their emotional state, and individuals with high emotional profile reported being less able to regulate their emotions, ruminate more and have deficits in emotion management than those in the other profiles. Based on the empirical evidence about the relationship between ANPS profiles and emotion regulation^25^, as well as theoretical considerations from attachment literature^45,46^, we explain the three latent profiles explored in our study in the context of affective typologies, that is we interpret the three groups as possible phenotypes of different emotion-regulation strategies. As individuals with Profile 1 had relatively high scores on both negative and positive scales, we suggest that this high emotional subgroup pattern might refer to the usage of hyperactivation strategies^46^. The cognitive and affective implications of hyperactivating strategies result in a tendency to intensify emotional responses, to heighten attentional focus on negative emotions to detect threats, especially in interpersonal contexts and to enlarge the potential negative consequences of these threats. These strategies encourage rumination and produce a self-amplifying cycle of distress. Hyperactivation strategies are usually associated with high scores on attachment anxiety^47^.

Individuals with Profile 3 scored low on both negative and positive scales, especially on the positive ones. This pattern of the Low emotional subgroup might refer to the usage of deactivation strategies. Deactivating strategies inhibit the experience of aversive emotional states and exclude these states from awareness, seem to block acknowledgment of negative experience and prevent the use of inner-state information in cognitive processing. This process causes negative affect to lose its power to influence cognitions. The primary goal is to avoid frustration, to maximize cognitive, affective, and physical distance from challenging and demanding situations. These strategies keep the person downregulated, foster personal disengagement and detachment, strive for self-reliance and independence^45^.

Individuals with Profile 2 had relatively low scores on the negative scales, and relatively high on the positive scales. According to Schwartz and Bohner^46^, positive affects signal that ‘all is going well’ and that one can explore unusual stimuli and associations in a relaxed and playful manner. This balanced strategy promotes openness to affective cues, facilitates creative exploration and broaden one’s perspectives, because it heightens confidence that one can deal effectively with uncertainty, novelty, and any confusion that the broadening of knowledge might create.

The first limitation of our study is that the empirical evidence is exclusively based on self-report ratings. The combination of applying different methods (e.g., self-report ratings and registration of neural responses, electrodermal activity or heart-rate variability etc.) can provide a more complex insight. Sample size can be also a limitation; however, it seems to be large enough. Second, confirmatory factor analysis is needed to analyze the efficacy of the models. Third, as LPA is an exploratory method, a more representative sample might show different profile models. Last, our results are limited to the BIG5 dimensions. Additional external constructs, for example, difficulties in emotion regulation, inter-and intrapersonal skills can further validate the ANPS profiles.

## Conclusions

We examined the emotional aspects of personality in a variable-centered and a person-centered approach. We identified a higher-order structure of personality including four super-traits and explored three latent profiles with different patterns of primary emotional traits. In sum, the person-centered approach and LPA methodology can offer a holistic way of personality research. Of course, replications are necessary to generalize the existence of the profiles, and further studies with clinical populations could be beneficial, as well. As the ANPS is a neurobiologically based instrument, mapping and validating the neurobiological bases of affective typologies (profiles) with brain imaging techniques could be fruitful.

### Supplementary Information

Supplementary Tables.

## Data Availability

On request from corresponding author.
