# Supplementary material for: Affective super-traits and/or individual patterns: a variable-centered and a person-centered approach of primary emotional aspects of personality
Source: Sci Rep. 2024 Feb 27;14:4787. doi: 10.1038/s41598-024-55371-4 (PMC10899169; doi:10.1038/s41598-024-55371-4)
Supplement: Supplementary file 1 — Supplementary Tables. [file 41598_2024_55371_MOESM1_ESM.pdf]

Supplementary information

Affective super-traits and/or individual patterns: A variable-centered and a person-centered approach to primary emotional basis of personality

Anita Deak[1*], Orsolya Inhof[1], Laszlo Nagy[1], Krisztina Csokasi[1]

[1]Institute of Psychology, Faculty of Humanities and Social Sciences, University of Pecs, Pecs, Hungary

Table of content

Supplementary Table 1. Summary of relevant biological-oriented theories in personality neuroscience 2

Supplementary Table 2. Gender differences of ANPS scales for the three profiles (Effect sizes report the Cohen's d). 4

Supplementary Table 3. Group comparisons of the ANPS scales for men and women, respectively. Effect sizes report the Cohen's d. 5

Supplementary Table 4. Descriptive statistics of the ANPS scales for the three latent profiles 6

Supplementary Table 5. Gender differences of BFI facets for the three profiles (Effect sizes report the Cohen's d). 7

Supplementary Table 6. Group comparisons of the BFI facets for men and women, respectively. Effect sizes report the Cohen's d. 8

Supplementary Table 7. Descriptive statistics of the BFI facets for the three latent profiles 9

Supplementary Table 1. Summary of relevant biological-oriented theories in personality neuroscience

Construct	Biological basis	Domains/Dimensions/Scales	Tool to Measure
Eysenck (Eysenck & Eysenck, 1985)	Arousability (ascending reticular activating system – ARAS)	Extraversion: reticulo-cortical circuit; high cortical threshold; Neuroticism: reticulo-limbic circuit; easily aroused by emotion-inducing stimuli; Psychoticism: negatively associated with serotonergic function, and positively with dopaminergic function	Eysenck Personality Questionnaire (EPQ)
The Five-Factor Model (Big5) (Goldberg, 1990)	Biological universality of BIG5 traits: the genetic factor structure is invariant across several samples.	Openness; Conscientiousness; Extraversion; Agreeableness; Neuroticism	Revised NEO Personality Inventory (NEO-PI-R)
Reinforcement Sensitivity Theory [1]	Neurobiological model with functional systems: BAS: dopaminergic system. FFFS: amygdala, hypothalamus, periaqueductal gray. BIS: septo-hippocampal system, amygdala. Anxiety=BIS sensitivity; Impulsivity = BAS sensitivity	Behavioral approach system (BAS): responds to rewarding cues. Fight-flight-freeze system (FFFS): responds to immediately threatening, punishing or frustrating stimuli. Behavioral inhibition system (BIS): responds to threatening or conflicting stimuli.	Behavioral Inhibition Scale and Behavioral Activation Scales (BIS/BAS)
The Alternative Five (Zuckerman, 2005)	Behavioral mechanisms (e.g., approach, inhibition) determined by multiple biological systems (e.g., neurotransmitters, hormones, enzymes) and contributes to multiple traits.	Sociability, Neuroticism-Anxiety, Aggression-Hostility, Impulsive Sensation-Seeking and Activity	Zuckerman–Kuhlman Personality Questionnaire (ZKPQ)
Biosocial model with neurotransmitter systems (Cloninger, 1997, 2004)	Neurotransmitter systems might be related uniquely to specific traits. NS: dopaminergic system. HA: serotonergic system; RD: norepinephrine system.	Temperament dimensions (genetically determined): Novelty-Seeking (NS), Harm-Avoidance (HA), Reward Dependence (RD). Character dimensions (determined by experiences): Persistence, Self-Directedness, Cooperativeness and Self-Transcendence	Temperament and Character Inventory (TCI)

Affective Neuroscience Theory (Panksepp, 1998)	Six traits reflecting distinct emotional systems	PLAYFULNESS, SEEKING, CARING, FEAR, ANGER, SADNESS (+ Spirituality)	Affective Neuroscience Personality Scales (ANPS)

Supplementary Table 2. Gender differences of ANPS scales for the three profiles (Effect sizes report the Cohen's d).

	Profile 1		Profile 2		Profile 3	
	t	Effect size	t	Effect size	t	Effect size
SEEK	8.13***	2.29	2.86#	.66	-6.29***	.85
CARE	8.25***	2.33	6.92***	1.61	-4.39***	-1.25
FEAR	5.72***	1.61	2.23	.52	-2.82#	-.80
PLAY	1.90	.54	-2.19	-.51	-5.01***	-1.43
ANGER	1.16	.33	-1.03	-.24	.69	.20
SADNESS	8.68	2.45	4.93***	1.14	-4.79***	-1.36

* p < .05; ** p < .01; *** p < .001; # .05 < p < .08

Supplementary Table 3. Group comparisons of the ANPS scales for men and women, respectively. Effect sizes report the Cohen’s d.

			P1 vs. P2		P1 vs. P3		P2 vs. P3	
	ANOVA F	Eta square	t	Effect size	t	Effect size	t	Effect size
Men								
SEEK	43.6***	.59	-1.42	-.41	-8.82***	-2.95	7.84***	2.54
CARE	12.9***	.30	-1.86#	-.54	-5.05***	-1.69	3.55***	1.15
FEAR	16***	.35	-5.64***	-1.63	-2.22*	-.74	-.2.75**	-.89
PLAY	31.6***	.51	4.35***	1.26	-3.83***	-1.28	7.85***	2.54
ANGER	2.66#	.08	.56	.19	-1.71	-.49	-2.11*	-.68
SADNESS	21.3***	.41	-6.28***	-1.82	-4.48***	-1.50	-.99	-.32
Women								
SEEK	41.5***	.32	-3.83***	-.63	6.34***	1.40	9.10***	2.03
CARE	53.0***	.38	-3.61***	-.59	7.71***	1.70	10.29***	2.29
FEAR	94.9***	.52	13.44***	2.20	7.64***	1.69	-2.33#	-.52
PLAY	31.4***	.26	-4.71***	-.77	4.25***	.94	7.68***	1.71
ANGER	38.3***	.31	8.63***	1.41	1.74	.38	-4.63***	-1.03
SADNESS	84.9***	.49	11.15***	1.83	10.29***	2.27	1.99	.44

* p < .05; ** p < .01; *** p < .001; # .05 < p < .08

Supplementary Table 4. Descriptive statistics of the ANPS scales for the three latent profiles

	Profile 1			Profile 2			Profile 3		
	Mean	SD	Min-max	Mean	SD	Min-max	Mean	SD	Min-max
Men									
SEEK	46.3	4.42	38-55	44.6	4.34	35-51	33.8	3.75	26-41
CARE	43.3	4.64	34-51	40.3	6.39	29-51	33.9	5.38	23-40
FEAR	42.0	7.57	29-54	30.5	6.41	20-43	36.8	7.37	19-50
PLAY	43.1	3.96	36-50	48.8	3.52	42-54	37.3	6.52	26-47
ANGER	36.0	8.13	21-48	32.0	8.88	16-56	37.5	5.86	30-48
SADNESS	41.5	6.3	28-53	31.6	5.04	20-41	33.3	4.86	22-41
Women									
SEEK	44.7	5.24	29-56	47.8	3.87	31-55	37.8	6.16	20-49
CARE	45.1	4.80	35-54	47.8	3.92	38-55	37.3	5.38	23-45
FEAR	47.4	4.77	38-56	33.6	6.94	16-49	36.8	7.95	14-51
PLAY	40.5	7.27	21-56	45.6	6.12	30-55	34.3	5.94	19-43
ANGER	39.9	6.56	21-56	30.5	6.62	19-45	37.4	7.24	25-51
SADNESS	45.3	4.37	36-56	37.0	4.72	22-47	34.9	4.78	24-45

Supplementary Table 5. Gender differences of BFI facets for the three profiles (Effect sizes report the Cohen's d).

	Profile 1		Profile 2		Profile 3	
	t	Effect size	t	Effect size	t	Effect size
Extraversion	2.58	.76	1.26	.29	-1.74	-.50
Agreeableness	3.47**	-98	4.16***	.96	-1.42	-.40
Conscientiousness	1.26	.35	2.26	.52	1.16	.33
Emotional instability	2.96*	.83	.95	.22	-2.61	-.74
Openness	3.99***	1.12	-.44	-.10	-3.52**	-1.00

* p < .05; ** p < .01; *** p < .001

Supplementary Table 6. Group comparisons of the BFI facets for men and women, respectively. Effect sizes report the Cohen's d.

	ANOVA F	Eta square	P1 vs. P2		P1 vs. P3		P2 vs. P3	
			t	Effect size	t	Effect size	t	Effect size
Men								
Extraversion	12.3***	.29	.46	.13	-4.16***	-1.39	4.70***	1.52
Agreeableness	4.64*	.13	-.26	-.08	-2.80**	-.94	2.66**	.86
Conscientiousness	2.77#	.08	.11	.03	-2.03*	-.68	2.19*	.71
Emotional instability	19.1***	.39	-6.01***	-1.74	-1.57	-.52	-3.75***	-1.22
Openness	12.8***	.30	-.30	-.09	-4.60***	-1.54	4.49***	1.45
Women								
Extraversion	17.1***	.16	-5.50***	-.90	-.14	-.03	3.91***	.87
Agreeableness	23.2***	.21	-5.41***	-.89	1.92	.42	5.89***	1.31
Conscientiousness	11.8***	.12	-4.79***	-.78	-2.51*	-.55	1.03	.23
Emotional instability	58.2***	.40	10.74***	1.76	4.84***	1.07	-3.08**	-.69
Openness	6.69**	.07	-.71	-.12	3.09**	.68	3.59***	.80

* p < .05; ** p < .01; *** p < .001; # .05 < p < .08

Supplementary Table 7. Descriptive statistics of the BFI facets for the three latent profiles

	Profile 1			Profile 2			Profile 3		
	Mean	SD	Min-max	Mean	SD	Min-max	Mean	SD	Min-max
Men									
Extraversion	28.6	6.02	18-39	29.5	5.25	19-38	21.1	5.07	11-28
Agreeableness	31.2	3.98	23-38	30.8	5.63	19-40	26.1	6.559	14-35
Conscientiousness	32.0	7.22	23-44	32.2	5.46	18-42	27.9	5.22	20-38
Emotional instability	27.1	5.18	21-37	18.4	5.13	9-29	24.5	4.45	17-32
Openness	40.2	5.80	28-48	39.7	7.05	24-50	30.7	5.08	24-42
Women									
Extraversion	25.6	6.54	12-37	31.2	5.53	18-40	25.8	6.69	14-39
Agreeableness	31.3	5.16	20-42	35.8	4.58	24-43	29.1	6.44	15-41
Conscientiousness	30.3	7.60	15-45	35.9	6.42	19-45	34.3	7.62	19-45
Emotional instability	29.0	5.48	16-40	19.3	4.71	11-28	23.1	7.13	8-36
Openness	38.2	6.12	23-50	39.0	7.30	21-50	33.5	7.70	18-46
